# Polymerase Discordance in Novel Swine Influenza H3N2v Constellations Is Tolerated in Swine but Not Human Respiratory Epithelial Cells

**DOI:** 10.1371/journal.pone.0110264

**Published:** 2014-10-16

**Authors:** Joshua D. Powell, Daniel Dlugolenski, Tamas Nagy, Jon Gabbard, Christopher Lee, Stephen M. Tompkins, Ralph A. Tripp

**Affiliations:** 1 Department of Infectious Diseases, Animal Health Research Center, University of Georgia, Athens, Georgia, United States of America; 2 Department of Pathology, College of Veterinary Medicine, University of Georgia, Athens, Georgia, United States of America; University of Rochester Medical Center, United States of America

## Abstract

Swine-origin H3N2v, a variant of H3N2 influenza virus, is a concern for novel reassortment with circulating pandemic H1N1 influenza virus (H1N1pdm09) in swine because this can lead to the emergence of a novel pandemic virus. In this study, the reassortment prevalence of H3N2v with H1N1pdm09 was determined in swine cells. Reassortants evaluated showed that the H1N1pdm09 polymerase (PA) segment occurred within swine H3N2 with ∼80% frequency. The swine H3N2-human H1N1pdm09 PA reassortant (swH3N2-huPA) showed enhanced replication in swine cells, and was the dominant gene constellation. Ferrets infected with swH3N2-huPA had increased lung pathogenicity compared to parent viruses; however, swH3N2-huPA replication in normal human bronchoepithelial cells was attenuated - a feature linked to expression of IFN-β and IFN-λ genes in human but not swine cells. These findings indicate that emergence of novel H3N2v influenza constellations require more than changes in the viral polymerase complex to overcome barriers to cross-species transmission. Additionally, these findings reveal that while the ferret model is highly informative for influenza studies, slight differences in pathogenicity may not necessarily be indicative of human outcomes after infection.

## Introduction

Influenza pandemics occur when an animal influenza virus to which humans have no or limited immunity acquires the ability through genetic reassortment or mutation to cause sustained human-to-human transmission leading to community-wide outbreaks. Influenza A viruses (IAV) have the capacity to exchange viral RNA segments when a host cell is co-infected with two IAV strains. IAV reassortment in swine is of global concern as swine support replication and reassortment of human, avian and swine IAV [1]–[3]. The term “mixing vessel” is often associated with IAV in pigs [4], [5] and is embodied by the widely circulating “triple reassortant of internal genes” (TRIG) constellation. The TRIG cassette comprises avian-origin PB2 and PA, human H3N2-origin PB1, and swine-origin M, NP and NS genes [6]. Initially identified in swine in 1998 [7], the TRIG-cassette is hypothesized to have a competitive advantage over other swine influenza virus (SIV) gene constellations [8], and it is thought that this feature helps maintain continued circulation in both H1 and H3 SIV lineages.

In 2009, five of six TRIG genes (with M gene from classical swine) emerged in the form of the human H1N1 pandemic (pdm09) [9]. Presently, pdm09 remains the dominant H1N1 circulating strain in humans emphasizing the need for continued pdm09 studies [10]. During the 2009 human pandemic, via reverse zoonosis, pdm09 was reintroduced back into swine [11] where 10 reassortant H3N2 SIV genotypes harboring one to six pdm09 genes were identified circulating in North American swine herds [12], [13]. Numerous SIV surveillance case-reports have also identified pdm09 genes within swine H1N1 and H1N2 subtypes on multiple continents [14]–[18]. The actual number of SIV constellations containing pdm09 segments is likely much higher, as globally swine surveillance is inconsistent and limited [19]. Given these concerns, determining which of the 256 possible reassortant combinations are compatible during co-infection between pdm09 and circulating endemic SIV strains is highly warranted, but remains largely uncharacterized.

While human infections with H1N1 and H3N2 SIV have been sporadically reported from 1990–2010 [20]–[22], commonly referred to as “variant” infections, in July 2011 the first H3N2 variant (H3N2v) containing a human-derived pdm09 segment (rH3N2p) was identified in humans [23]. There were 320 confirmed cases of H3N2v documented over this period, where many of these cases were linked to direct contact with swine [24]. It was estimated that that total number of H3N2v related infections exceeded 2000 cases [25].

Whole-genome sequencing of 14 human H3N2v infections has identified the pdm09 M gene consistently occurring within swine H3N2-TRIG viruses [26]. The pdm09 M gene is linked to increased transmissibility [27] [28]; therefore, this segment within H3N2v may represent a critical determinant for swine-to-human transmission. To address the reassortment potential of pdm09 and swine H3N2-TRIG viruses to combine as an H3N2v constellation, and generate infectious (compatible) or non-infectious (incompatible) pdm09-based reassortants, swine cells from a porcine kidney epithelial cell line (PK-1) and a primary normal swine bronchoepithelial cell line isolated from euthanized pigs [29], [30] were co-infected with the pdm09 strain (A/California/04/09) and a recently characterized H3N2-TRIG virus (A/Sw/PA/62170-1/2010). This swine H3N2-TRIG isolate was selected as it has established virulence and contact transmissibility in pigs [31]. Furthermore, the HA gene is of cluster-IV lineage, similar to H3N2v human cases [8], and over 99% identical to an isolate found in a human swine farmer in Ontario, Canada in 2005 [22], given its potential for human infection.

In this study, examination of 750 influenza virus isolates from plaques in NSBE cells or PK-1 swine cells co-infected with pdm09 and swine H3N2 viruses showed reassortment for the 6 IAV segments tested (NA, HA, NS, M, PA, and PB1). Later, NP and PB2 IAV segments were deduced for a subset of reassortants. Interestingly, the polymerase acidic (PA) segment of pdm09 occurred within seven genes of H3N2-TRIG (swH3N2-huPA), and this feature was shown to have both a reassortment and replication advantage in the swine cell lines which is consistent with its known functions in the RNA polymerase complex having both endonuclease and cap binding function [32], [33]. A second protein encoded within the PA segment, PA-X, is synthesized as a ribosomal frame-shift, and has been shown to modulate virulence and host innate responses and suppress host protein synthesis [34], [35]. Given the critical functions of the PA gene, and the continued circulation of pdm09 PA viruses in both swine and humans [36], it was important to determine if the PA gene represents a barrier to viral fitness in swH3N2-huPA reassortants, and determine if differences in replication could be linked to differences in the antiviral host response comprising type I and III interferons (IFNs). Interestingly, replication of one reassortant virus harboring both PA and PB1 pdm09 polymerase genes (swH3N2-huPA-PB1) was attenuated in swine cells and human cells, suggesting that PA-PB1 is a feature that may govern attenuation at the innate cell level in swine and human epithelial cells. Ferrets infected with swH3N2-huPA isolates had increased lung pathogenicity compared to parent viruses confirming the role of the pdm09 PA, and these isolates induced high expression of IFN-β and IFN-λ genes in human but not swine cells. These findings provide evidence that genetic reassortment resulting from co-infection of swine with endemic SIV and human IAV strains may result in novel reassortant viruses with varied degrees of pathogenicity attributed to in part by the the polymerase complex.

## Materials and Methods

### Ethics statement

All ferret experiments were performed in accordance to the national guidelines provided by the “The Guide for Care and Use of Laboratory Animals” and The University of Georgia Institutional Animal Care and Use Committee (IACUC). The animal use protocol A2012 06-025-Y2-A2 was approved by the University of Georgia IACUC.

### Viruses

The H3N2 swine isolate A/Sw/PA/62170-1/2010 [swH3N2] was acquired from the National Veterinary Services Laboratory (NVSL). Pathogenesis, transmission and genetic sequence characterization for this swH3N2 in pigs has been previously established [31]. The human H1N1 virus, A/California/04/2009 (pdm09), was provided by the Center for Disease Control and Prevention (Atlanta, GA) and has been previously characterized [37].

### Cells

Viruses were propagated in Madin-Darby Canine Kidney (MDCK; ATTC #CCL-34) cells. The PK-1 cell line was acquired from ATCC (#CL-101). The isolation, culturing and differentiation of primary NSBE has been described previously [30]. NSBE cells were verified by University of Georgia Veterinary Medical Diagnostic Laboratory (VMDL) as PCR-negative for influenza, circovirus and porcine reproductive respiratory syndrome virus before use. Primary normal human bronchoepithelial (NHBE) cells were purchased (Lonza CC-2540). Both NSBE and NHBE cells were cultured at air-liquid-interface.

### Co-infection and plaque isolation

Co-infection of swine cells was performed at MOI = 3 in infection media (DMEM, 1 mM L-glutamine, 0.5 µg/mL trypsin). The cell supernatant was collected at 18 h post-infection (hpi) and the PFU/mL determined by plaque assay as previously described [38]. PK-1 and NSBE cell supernatants were added to confluent MDCK cells in 15-cm^2^ tissue culture dishes for 1 h in DMEM, washed 2X PBS, and overlaid with infection media supplemented with 0.225% sodium bicarbonate and 2% agarose. Serial dilutions were optimized to ensure adequate spacing between virus plaques (<100 plaques). Plaques isolated from agar were amplified in 48-well plates using the NSBE cells or PK-1 cells for 48 h before RNA extraction and multiplex qPCR analysis. Additionally, select reassortants were propagated in MDCK cells in the presence of 1 µg/ml trypsin to generate viral stocks for downstream experiments. After propagation in MDCK, the PCR Ct values for viral stocks were verified to confirm that there was no mixed infection.

### qPCR multiplex

Supernatants from virus-infected PK-1 and NSBE cells cultured in 48-well plates of were RNA purified using an RNeasy Mini-kit (Qiagen). 500 ng of RNA was used for cDNA synthesis with a Verso cDNA kit (Thermo Scientific) using random hexamer primers according to manufacturer's instructions. cDNA reactions were diluted 10-fold and used as template for qPCR analysis. Multiplex qPCR primer/probe sequences and their location within genome segments of SwH32-PA [TaxID: 938271] and huH1N1-CA [TaxID: 641501] are noted in Fig. S1. After primers were initially verified for specificity by using SYBR GreenER Supermix PCR (Life Technologies), Quencher probes Iowa Black FQ-Cy5 and ZEN-double-quenched probes specific for 6-FAM and HEX, (Integrated DNA Technologies) were implemented in a 3-plex reaction using a Quanti-Tech Multiplex PCR kit (Qiagen). cDNA from each corresponding plaque was analyzed in 4 separate reactions specific for either swine or human HA-NA-NS1 and swine or human PB2-PA-M. Ct values for swH3N2 and huH1N1 positive controls isolated as described above and the schematic for qPCR analysis is shown in Fig. S2, confirming the specificity of the 2×3-plex six gene assay. The remaining two influenza genes, PB1 and NP for select reassortants were deduced by SYBR GreenER supermix PCR (Life Technologies) (Fig. S1,bottom). All qPCR reactions were carried out in an Agilent Mx3005P qPCR instrument with an initial 5 min denaturation, and 40 cycles of denaturation at 95°C, 15 sec of annealing at 57°C, 15 sec, and 15 sec of extension at 72°C.

### Co-infection studies

Supernatants (1 µL) from the 24-well virus isolates at the corresponding MOI were used to co-infect PK-1 cells or NSBE cells for 48 h. For each respective passage, the cells were washed 2X in PBS, and the infections proceeded for an additional 48 h. This was repeated for all subsequent passages. At passage 8, a multiplex qPCR was used to determine the six influenza gene segments. The additional two genes, PB1 and NP, were determined by SYBR GreenER Supermix qPCR (Life Technologies).

### Viral kinetics

Virus stocks of selected reassortants were analyzed in PK-1 cells, MDCK cells, undifferentiated normal swine bronchoepithelial cells, fully differentiated normal swine bronchoepithelial (dNSBE) cells, and fully-differentiated normal human bronchoepithelial (dNHBE) as described [30], [39] at an MOI = 0.01 for 24, 48, 56 and 72 hpi. All viral titers were determined by the 50% tissue culture infectious dose (TCID_50_) assay [40]. Peak titers were confirmed by plaque assay using 1.2% Avicel, and by TCID_50_ assay. The dNHBE and dNSBE cell infections were performed using DMEM without exogenous trypsin at an MOI = 0.01, the cells washed 3X in PBS, and infections allowed to progress at the air-liquid interface. Apical titers were determined by gently washing cells 2×500 µL PBS. Basolateral titers were directly deduced from primary growth media.

### Plaque morphology and size

Ten-fold serial dilutions of supernatant from virally infected cells was overlaid with 2% agarose in 1X overlay media for 4 days and stained with crystal violet [41]. Selected plaques, totaling 10–20 plaques/well from 2–3 representative wells, were scanned using a standard flatbed Hewlett Packard Inkjet scanner. Relative plaque diameters were measured using Photoshop 5.0 software.

### qPCR gene expression in dNHBE and dNSBE cells

Taqman qPCR primer probes for determining the innate signaling response were purchased from Life Technologies and used per the manufacturer suggestion. RNA extraction and cDNA synthesis reagents were performed as described above for qPCR multiplex. 500 ng of total RNA was used in cDNA synthesis and diluted to a final concentration of 2.5 ng for each qPCR reaction. Primer-probes were commercially prepared from Life Technologies are as previously noted [30].

### Ferret studies

Outbred 0.8–1.0 kg juvenile female ferrets (Triple F Farms, Sayre, PA), were tested and determined to seronegative by hemagglutination inhibition assay to influenza virus. Only seronegative ferrets were used for the studies. Prior to infection, a subcutaneous implantable temperature transponder (BioMedic Data Systems, Seaford, DE) was used to monitor body temperature. Ferrets were anesthetized with isoflurane and intranasally inoculated with 0.2 ml of virus (0.1 ml per nostril) at 10^6^ PFU/ml totaling 2×10^5^ PFU. Weights and temperatures were monitored every other day for 8 days pi, and at day 15 pi prior to euthanasia. Nasal washes were obtained from ferrets on days 2, 4, 6, and 8 days pi. Ferrets were anesthetized with ketamine (30 mg/kg), and nasal washes isolated as described previously [37]. Both TCID_50_ and plaque assays (PFU/ml) were used to confirm viral load in nasal washes. Five ferrets (n = 5) were used for each experiment.

### Histopathological examination of ferret tissues

In addition to ferret nasal wash studies, three ferrets per virus were used to determine histopathology at day 3 pi (2×10^5^ PFU) with swH3N2, pdm09 or swH3N2-huPA virus, and two ferrets were mock-infected with PBS. At day 3 pi, ferrets were euthanized and the head and lungs fixed in 10% buffered formalin for histological examination. Bone was decalcified for 14 days prior to nasal turbinate analysis. Both hematoxylin and eosin (H&E) staining and immunohistochemical (IHC) analysis was performed as previously reported [37]. Briefly, lung and nasal turbinates were embedded in paraffin wax, sectioned 4–5 µm thick, and placed on glass slides. Tissues were examined by IHC and using H&E staining to detect lesions consistent with viral infection. For IHC, primary mouse anti-NP influenza mouse monoclonal antibody (ATCC; *H16*-*L10*-*4R5*) was used at 5 µg/ml.

### Immunofluorescence co-localization analysis

Co-localization studies in dNSBE and dNSBE cell studies were performed as previously described [39]. Briefly, cells were stained for α2,6 or α2,3 sialic acids, permeabilized, and stained with anti-NP influenza antibody [ATCC; *H16*-*L10*-*4R5*] and AlexaFluor 488 (Molecular Probes). Images were obtained with a Zeiss LSM 710 confocal microscope.

### Statistical analysis

Statistical analysis was performed using GraphPad Prism 5 software. One-way ANOVA analysis was performed using GraphPad Prism software with p<0.05 as indicated by an asterisk (*).

## Results

### Swine H3N2-TRIG viruses containing the pdm09 PA gene have a reassortant advantage

**Figure 1:**
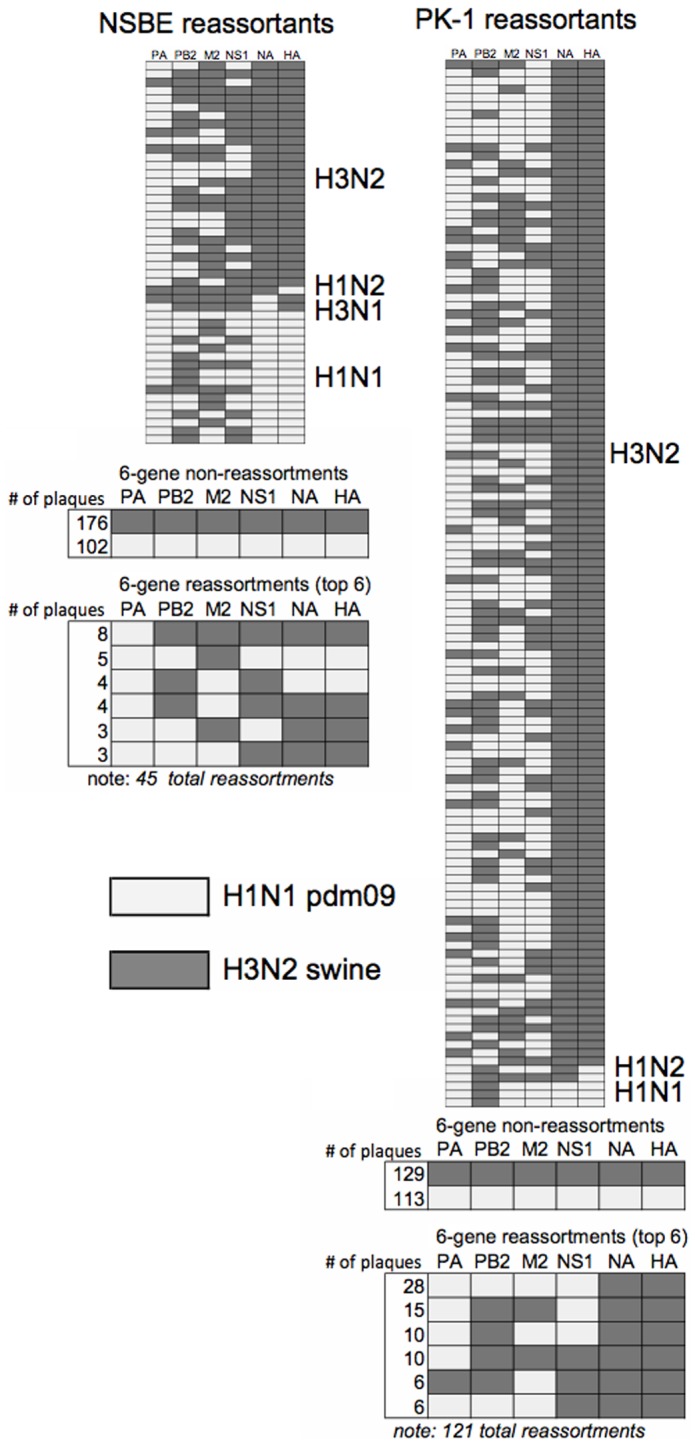
Reassortment outcomes following human H1N1 pdm09 and swine H3N2 co-infection. Segments originating from pdm09 origin (light colored) and swH3N2 (dark-colored) are shown after co-infection in NSBE cells (left) and PK-1 cells. Primer and probes specific to parental strains pdm09 and swH3N2 gene segments were used and from left to right in the matrix correspond to PA, PB2, M, NS, NA and HA. All reassortant plaques identified are categorized by HA and NA backbone type. In total, 121 reassortants for PK-1 cells and 45 reassortants for NSBE cells were evaluated. The number of virus plaques showing 6-gene non-reassortment and the top 6 reassortant types are summarized below profile matrix.

A 6-gene multiplex qPCR assay for segments PA, PB2, M, NS, NA and HA was utilized to delineate the parent segment for selected plaques. In total, 363 of 408 PK-1 cell plaques, and 323 of 351 NSBE cell plaques were distinguished as to the origin of the parent viral segment (Fig 1). While a majority of plaques were not reassortants for the six genes analyzed, 45 of 323 NSBE cell plaques (14%; Fig. 1, left), and 121 of 363 PK-1 cell plaques (33%; Fig. 1, right) were reassortants. qPCR multiplex profiling confirmed the PA segment of pdm09 was the most frequent reassortant gene which was identified in 93 of 121 PK-1 cell reassortants, in 39 of 45 NSBE cell reassortants, and was the primary gene detected in the swine H3N2 HA/NA backbone. The top six reassortant constellations identified from plaques is summarized in Figure 1. Interestingly, only five PK-1 cell-derived reassortants were identified that contained HA or NA genes of pdm09 origin. While reassortment frequency was lower in NSBE cells compared to PK-1 cells, a higher degree of diversity in genotypes was derived from NSBE cells implying fundamental differences in co-infection outcomes for swine cells of kidney origin (PK-1 cells) compared to those isolated as primary lung respiratory epithelial cells. Nonetheless, both swine cell lines revealed preferential pdm09 PA gene incorporation within the swine H3N2 backbone. Of note, due to the high nucleotide similarity of NP and PB1 for parent strains, determining the parental viruses in a high-throughput screening approach was not viable using quencher probes. However, subsequent SYBR green qPCR primers were designed to target a more unique region of PB1 and NP (Fig. S1) which was specific, thus both qPCR approaches were used to determine all eight gene segments for reassortants reported.

### pdm09 PA gene reassortment

**Figure 2:**
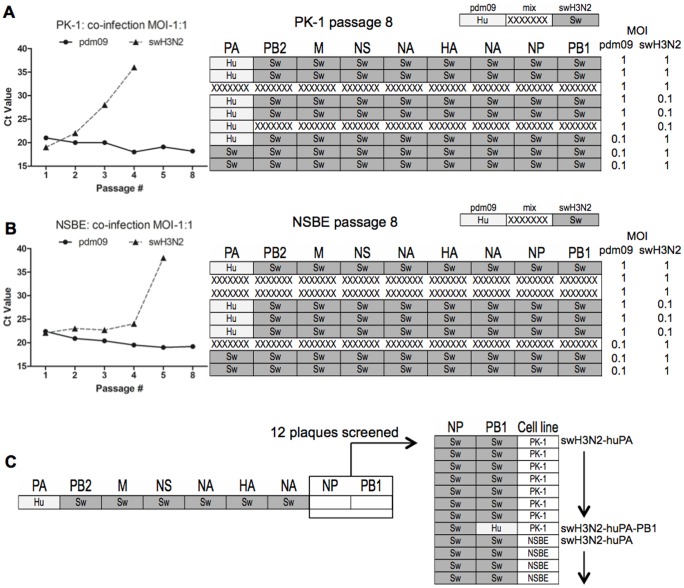
Reassortment findings after eight serial passages. Co-infection was performed in (A) PK-1 cells and (B) NSBE cells. While pdm09 and swH3N2 parent viruses were evident the only reassortant constellation identified was the PA segment of human pdm09 (light colored, “Hu”) within seven gene segments from swH3N2 (dark colored, “Sw”). Mixed gene segments with Ct values <8 are depicted as hash marks. Serial passaging was done in triplicate with a starting MOI of either 1.0 or 0.1 for each virus as indicated. To the left of (A) PK-1 cells and (B) NSBE cells is the kinetics of PA acquisition and loss for a MOI = 1∶1. Depicted is the first replicate shown from matrix. (C) 12 plaques with PA gene of pdm09 and 5 segments of swH3N2 from reassortant studies (Fig. 1) were subjected to qPCR do delineate the final two segments, NP and PB1. Depicted on right, 11 of 12 reassortants were determined to be swH3N2-huPA, and 1 of 12 reassortants to be swH3N2-huPA-PB1.

To determine if a dominant reassortant genotype occurred after co-infection with pdm09 and swH3N2 viruses in swine cells, a multiplex qPCR approach (Fig. 1) was combined with SYBR green qPCR for NP and PB1 that enabled all viral segments to be determined. An equivalent starting MOI = 1∶1, or 10-fold bias (MOI = 1.0∶0.1 or 1.0∶0.1) for pdm09 and swH3N2 was used to infect PK-1 cells (Fig. 2A) and primary NSBE cells (Fig. 2B). Although in limited cases a dominant genotype for a given gene segment could not be definitively identified by qPCR, and were identified as multiple viral isolates are denoted as “mixed” in Fig. 2 (X-dashed), there was a high propensity for human “hu” pdm09 PA incorporation within swine “sw” H3N2 backbone (Fig. 2B). Subsequently, viral RNA from the supernatant of MOI = 1∶1 cultures was probed to determine pdm09 PA gene acquisition and swH3N2 PA gene loss for all eight serial passages. It was confirmed that pdm09 PA was acquired between passages 3–5 (Fig. 2, left). Of note, cDNA from 12 plaques isolated (Fig. 1) having a gene constellation from swH3N2 and pdm09 PA were also screened for incorporation of the other polymerase genes and NP (Fig. 2C). Consistent with passaging studies (Fig. 2A, B), only one-of-twelve isolates had additional pdm09 polymerase segments corresponding to the PB1 of pdm09 virus (Fig. 2C). Of note, neither PB1 nor PB2 genes from pdm09 were present in swH3N2 after passaging Collectively; these results indicate that the PA gene of pdm09 provides a reassortment advantage without the preferential need of pdm09 segments PB1 and PB2. Given these findings, swH3N2-huPA, the single swH3N2-huPA-PB1 reassortant (Fig. 2C) and the parent viruses, swH3N2 and pdm09, were further studied.

### The pdm09 PA gene facilitates replication in swine cells

**Figure 3:**
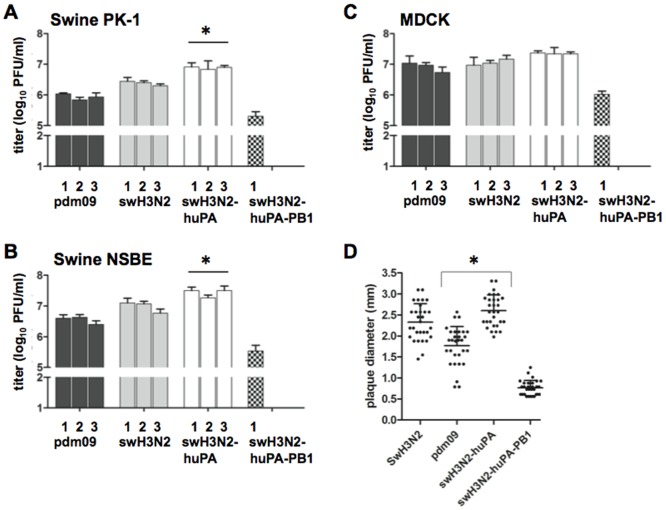
Peak virus titers from plaque isolates with pdm09, swH3N2, swH3N2-huPA, and swH3N2-huPA-PB1 gene constellations. The cell lines used are: (A) PK-1 cells, (B) NSBE cells, and (C) MDCK cells. Significant (p<0.05) differences in viral titer for swH3N2-huPA and both parental strains is indicated by an asterisk (*). Of note, only one swH3N2 reassortant with huPA-PB1 genes was identified (Fig. 2C). All infections used a MOI = 0.01. (D) Plaque diameter were measured 96 h pi in 2% agarose overlay solution. Cumulative plaque total measured from 2–3 separate six-well plates with 10–20 total plaques dilutions per well. * denotes significant difference between pdm09 and swH3N2-huPA.

Reassortment profiles (Fig. 1) and replication trends (Fig. 2A, B) infer that the swH3N2-huPA gene constellation improves viral fitness and replication. Thus, the replication kinetics of swH3N2-huPA compared to its parent strains was determined for the three virus isolates. In addition, the swH3N2-huPA-PB1 isolate (Fig. 2C) was also evaluated. As anticipated, all three swH3N2-huPA isolates exhibited higher peak virus titers in PK-1 cells (Fig. 3A) and undifferentiated NSBE cells (Fig. 3B) compared to parent strains. In MDCK cells, although not significant, higher peak virus titers trended for swH3N2-huPA compared to the parent strains (Fig. 3C). The swH3N2-huPA-PB1 reassortant was appreciably attenuated in all three cell lines (Fig. 3A, B, C), a feature that may explain the limited detection of this constellation after passaging (Fig. 2C). Agarose plaque assays used to determine plaque morphology revealed that the acquisition of the pdm09 PA gene for swH3N2-huPA resulted in plaque morphology similar to the swH3N2 virus. This finding was more pronounced when the agarose concentration was increased to 2%, a feature that allows for larger plaque size and was revealed for swH3N2-huPA compared to swH3N2 viruses (Fig. 3D). These findings suggest there is enhanced replicative fitness for swH3N2-huPA virus compared to swH3N2-huPA-PB1 virus, or the parental strains. Notably, a swH3N2-huPB2 constellation was not detected in the initial screen (Fig. 1), subsequently 22 virus plaques which had 6 gene segments of swH3N2 (Fig. 1) were screened for pdm09 PB1 and NP within swH3N2, but neither pdm09 PB1, PB2 or NP was detected within the seven remaining swH3N2 segments.

### swH3N2-huPA reassortants have increased pathogenicity in a ferret model

**Figure 4:**
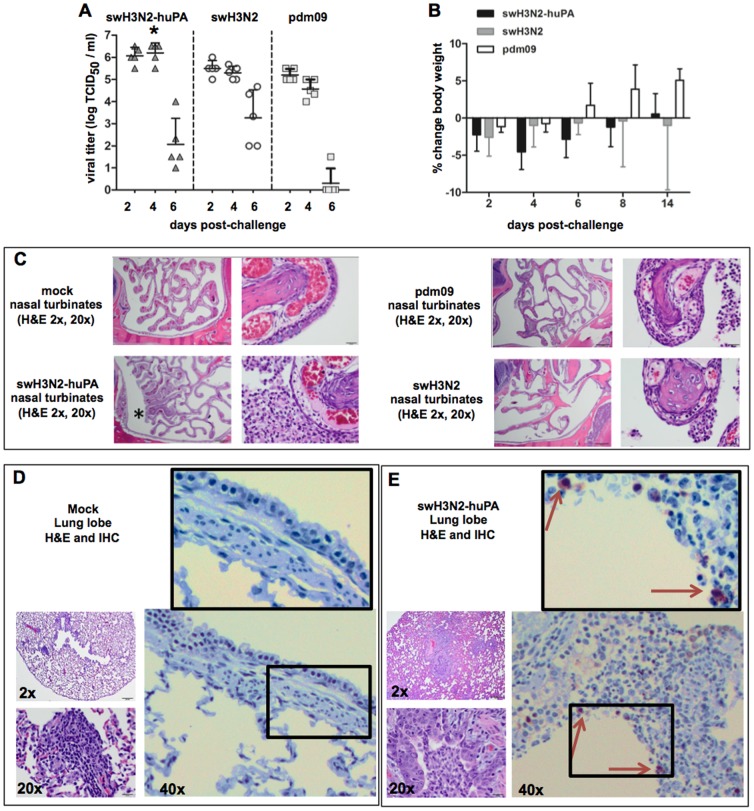
swH3N2-huPA infection is linked to increased nasal virus titers and pathology at early time points post-infection in ferrets (A). Virus titers from nasal washes indicate an increased viral load for swH3N2 at day 2 and 4 pi. An asterisk (*) denotes a significant (p<0.05) difference between swH3N2-huPA and both parental strains at day 4 pi. (B) Percent change in body weight over the course of infection as a measure of morbidity at day 4 pi following swH3N2-huPA infection. (C). Representative H&E images from nasal turbinates at 3 days pi. 2× magnification (left) and 20× magnification for mock, swH3N2-huPA and parent viruses. Asterisks show the region of magnification for swH3N2-huPA, and highlight the increased rhinitis in nasal turbinates and accompanying cell infiltration. Representative lung images following mock (D) and swH3N2-huPA (E) infection at day 3 pi showing H&E at 2X and 20X (left) and IHC stains at 40X. Arrows denote NP-positive cells with magnification of boxed area for viewing clarity. All ferrets excluding mock animals were infected with 2×10^5^ PFU.

Based on the findings showing increased replicative fitness for swH3N2-huPA virus (Fig. 3), and the reassortment profiles and genotyping (Fig. 1 and 2), the ferret model was used to assess the pathogenicity of the swH3N2-huPA virus (Fig. 4). Nasal inoculation of ferrets with pdm09, swH3N2 or swH3N2-huPA viruses revealed increased nasal wash virus titers for swH3N2-huPA at day 2 pi, and considerably increased virus titers at day 4 pi compared to parent virus titers (Fig. 4A). Morbidity was assessed by the percent body weight change during the course of infection. At day 4 pi, although not significant (p>0.05), there was a trend toward increased body weight loss in ferrets infected with swH3N2-huPA (Fig. 4B). Notably, at day 4 pi, there was a thick nasal discharge in 4 of 5 ferrets infected with swH3N2-huPA, but only 2 of 5 swH3N2-infected ferrets, and 1 of 5 pdm09-infected ferrets displayed substantial nasal discharge. At day 6 pi, clinical symptoms of infection had dissipated in all ferrets cohort. Hematoxylin and Eosin (H&E) pathology studies were performed at day 3 pi in ferrets infected with pdm09, swH3N2 or swH3N2-huPA viruses (Fig. 4C). The findings revealed increased rhinitis and inflammation of the mucous membrane in nasal turbinates of swH3N2-huPA infected ferrets compared to ferrets infected with parent viruses, as well as elevated levels of infiltrating cells (Fig. 4C, asterisk), and thinning of the epithelial cell lining. At day 3 pi, 2 of 3 swH3N2-huPA infected ferrets had detectable influenza NP antigen by IHC, and pulmonary cell infiltration as assessed by H&E staining (Fig. 4D vs. 4E). In contrast, very few NP-positive cells were detected in the lungs ferrets infected with swH3N2 virus (1 of 3 ferrets), or ferrets infected with pdm09 virus (1 of 3 ferrets) at day 3 pi. The pathology findings (Fig. 4C, D, E) and the enhanced nasal virus shedding observed at day 2 and 4 pi (Fig. 4A), suggest that pdm09 PA gene reassortants cause increased pathogenesis in a ferret model.

### swH3N2-huPA viruses replicate efficiently in differentiated swine respiratory epithelial cells

**Figure 5:**
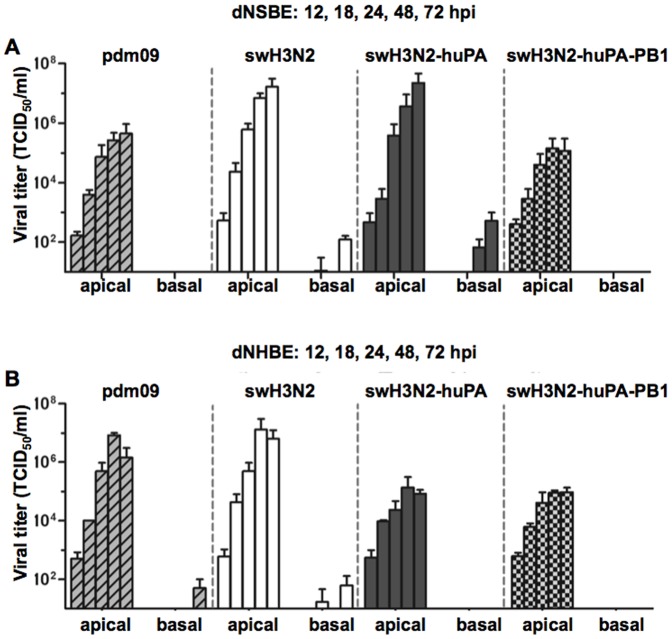
Kinetics of parent and reassortant virus replication in dNSBE and dNHBE cells. Replication kinetics in (A) swine dNSBE cells and (B) dNHBE cells at air-liquid interface. Viruses were infected on the apical side (left) and assessed for the ability to migrate through both the mucus layer and the polarized multi-layer epithelial architecture to the basolateral side [4].

A majority of influenza pathogenesis studies are performed in the ferret model. Although these studies provide insight, the findings do not fully reflect the range of disease associated in humans infected with influenza virus. In contrast, primary normal bronchial epithelial cells from humans or swine can provide a surrogate model of lung disease to help elucidate some of the mechanisms of disease pathogenesis in the airways [30], [39], [42]. Primary normal swine bronchoepithelial cells and primary normal human bronchoepithelial cells cultured at air-liquid interface polarize and form a three dimensional cell architecture comparable to typical lung architecture [30], [39], [42]. During differentiation of NSBE (dNSBE) and NHBE cells (dNHBE), these cells form tight junctions and are capable of secreting mucus. dNHBE and dNSBE cells express sialic acid (sias) receptor α2–6, and lower levels of α2–3 sias [39], [43], [44]. To determine if differences in Type I or III IFNs by dNSBE and dNHBE cells responding to IAV infection were a barrier restricting cross-species infection, and/or reassortment, comparative studies were performed. The results showed that H3N2-huPA-PB1 replication was attenuated in both cell lines, and swH3N2-huPA was attenuated in dNHBE cells but not dNSBE cells (Fig. 5A vs. Fig. 5B). These findings indicated innate antiviral host response differences between swine and human epithelial cells.

The kinetics of swH3N2, pdm09, swH3N2-huPA and swH3N2-huPA-PB1 replication in dNSBE cells (Fig. 5A) were consistent with that in PK-1 cells and undifferentiated NSBE cells (Fig. 3A, B). This was evident for swH3N2-huPA by the level of virus isolated from apical washes, and by detection in the basolateral media. These findings support the concept of enhanced replication fitness for swH3N2-huPA compared to the parent strains in dNSBE cells. Interestingly, when the viruses were assessed for replication in dNHBE cells, there was an approximate 2-log attenuation of replication for the reassortant viruses compared to parental strains (Fig. 5B), and no virus was isolated from the basolateral media. These findings show that neither pdm09 PA alone, or in combination with pdm09 PB1 in a swine H3N2 context enhances replicative fitness in human dNHBE cells.

**Figure 6:**
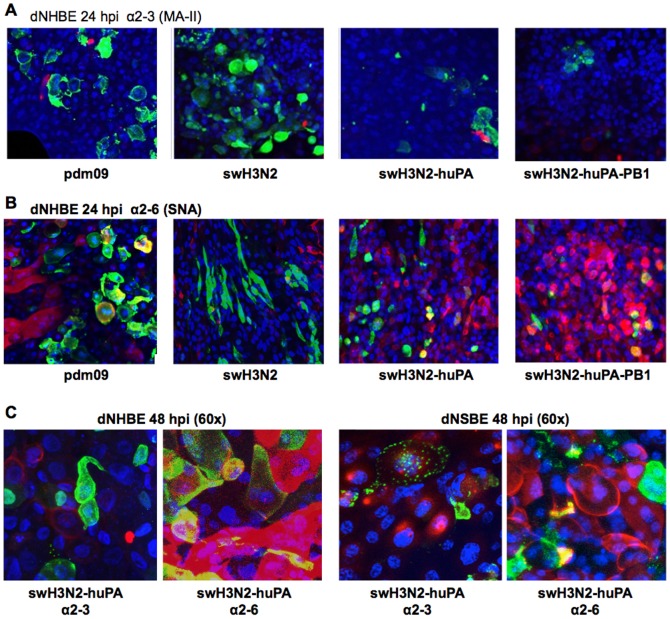
Immunofluorescence co-localization studies of virus and sialic acid expression on dNHBE cells. To determine co-localization of virus with sialic acid expression, cells were examined for either (A) α2–3 sialic acid by MAA-II lectin stain (red) or (B) **α**2–6 SNA lectin stain (red). DAPI was used for nuclear staining (blue). Yellow indicates co-localization of the NP with sialic acids. (C) 60× magnification of swH3N2-huPA reassortant showed similar localization profiles as other viruses in dNHBE cells (left) and dNSBE cells [4] revealing minimal to no localization (yellow) in α2–3 stained cells versus more noticeable NP co-localization with α2–6 sialic acid.

Given the differences in virus replication in dNSBE cells compared to dNHBE cells some of the features linked to virus tropism were investigated. The level of α2–3 and α2–6 sias expression on dNHBE and dNSBE cells was determined (Fig. 6). Immunofluorescence co-localization studies of α2–3 sias (Fig. 6A) and α2–6 sias (Fig. 6B) on dNHBE cells following swH3N2-huPA infection showed no substantial difference in expression among the sias as indicated by the level of sias co-localization (yellow; Fig. 6). As expected from earlier related studies [39], swH3N2-huPA virus infection co-localized with α2–6 sias (Fig. 6B, C), but was not exclusively reliant on α2–6 expression for infection based on influenza NP staining and α2–3 and α2–6 sias staining of dNHBE and dNSBE cells. Consistent with the replication kinetics (Fig. 5), levels of influenza NP (green staining) appeared less abundant at 24 h pi for reassortants compared to parental viruses post-infection (MOI = 0.01) of dNHBE cells (Fig. 6A, B). Attenuation of virus replication was also confirmed at 48 h pi in dNHBE cells (representative images in Fig. S3), indicating that viral attenuation (Fig. 5) may relate to NP abundance as determined by immunofluorescence (Fig. 6B, S3). Higher resolution (60X) images for swH3N2-huPA infection of dNHBE or dNSBE cells showed similar tropism based on immunofluorescence and co-staining of α2–3 and α2–6 sias despite the scant cells infected (Fig. 6C). Of note, >10% of dNSBE cells were positive for α2–3 sias (Fig.6C, right), while <5% of NHBE cells expressed α2–3 sias (Fig. 6C, left). Interestingly α2–3 sias staining for both dNSBE and dNHBE cells showed patchy expression (Fig. 6C). These findings indicate that the differences between swH3N2-huPA and parent virus replication in normal fully differentiated swine or human bronchoepithelial cells is not based on differences in cell tropism linked to α2–3 and α2–6 sias expression.

**Figure 7:**
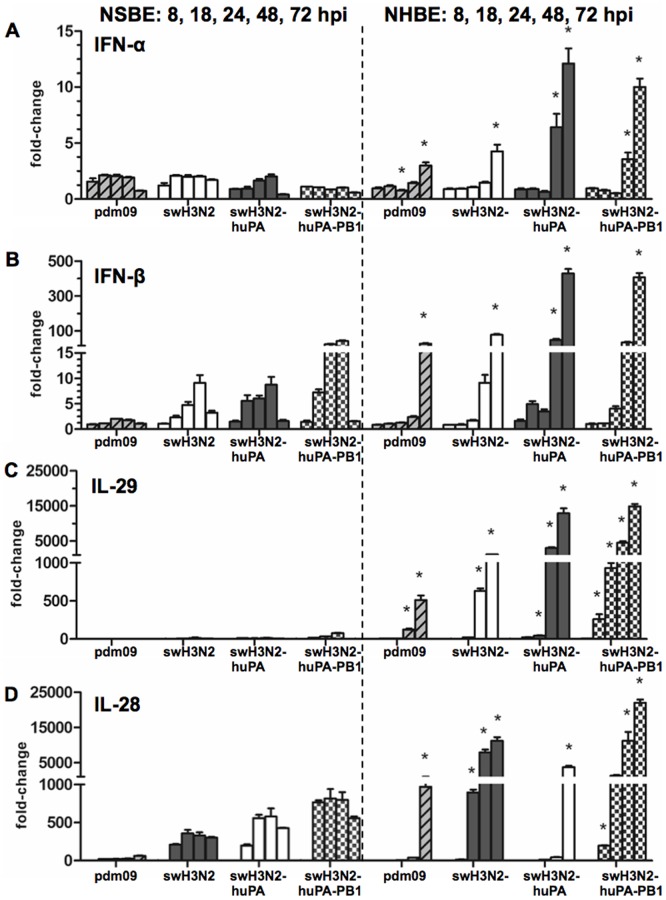
qPCR analysis of the type I and III IFN response in dNSBE and dNHBE cells. The qPCR fold-changes at 8 h, 18 h, 24 h, 48 h, and 72 h pi are shown for virus-infected dNSBE (left) and NHBE cells [4]. Shown is type I interferons (A) IFN-α (B) IFN-β and type III interferons (C) IL-29 (D) IL-28. Time-points that were significant (*  =  p<0.05) by one-way ANOVA comparing swine and human gene expression are indicated. Fold-changes and standard deviations were calculated from Ct values run in quadruplicate relative mock and the housekeeping gene HPRT1.

As differences in α2–3 and α2–6 sias expression did not solely account for differences in swH3N2-huPA or parent virus replication in dNHBE and dNSBE cells (Fig. 6), differences in the expression of interferon (IFN) IFN-α and IFN-β was explored (Fig. 7). Both type I interferon and type III IFN-λ genes (IL-28 and IL-29) are antiviral and can modulate virus replication [45]. qPCR analysis showed that infection of dNSBE with the parental viruses does not appreciably induce IFN-α (Fig. 7A), IFN-β (Fig. 7B) or IFN-λ IL-29 (Fig. 7C) expression at early time points of infection which is in agreement with previously qPCR findings in dNSBE cells infected with avian, swine and human IAV strains [30]. However, IL28, the only other detectable type III interferon in swine [46], was highly up-regulated at the transcript level in NSBE, a novel and yet unreported finding regarding IAV infection in swine (Fig. 7D).

**Figure 8:**
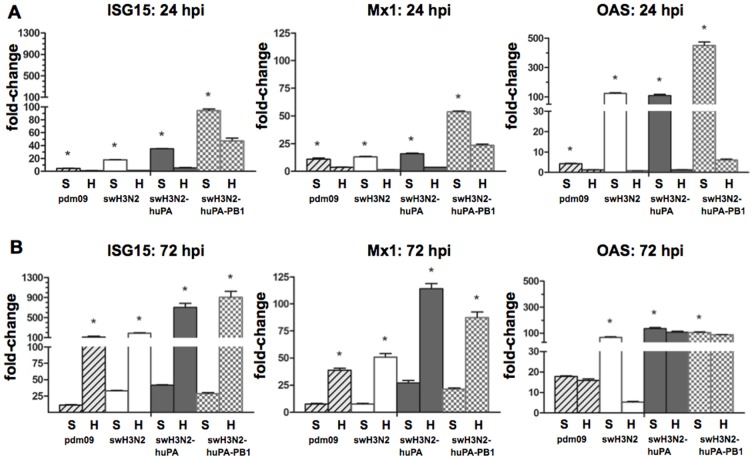
qPCR analysis of IFN-stimulated genes (ISGs). Expression of ISG15 (left), MX1 (middle) and OAS1 [4] was determined at (A) 24 h and (B) 72 h pi where “S” denotes swine dNSBE and “H” denotes human dNHBE cells. Fold-changes and standard deviations were calculated from Ct values run in quadruplicate relative mock and the housekeeping gene HPRT1. Time points that were significant between NSBE and NHBE cells are noted (*  =  p<0.05).

Comparison of type I and III IFN expression in dNSBE and dNHBE cells following infection with reassortant and parent viruses showed modest IFN-α induction (Fig. 7A) at 48 h and 72 h pi for reassortant viruses in human but not swine cells. Notable are the findings in dNSBE following swH3N2-huPA-PB1 infection, and to a lesser extent, the findings following swH3N2-huPA infection which showed increased IFN-β (Fig. 7B), IFN-λ (Fig. 7C) and IL-28 (Fig. 7D) expression at earlier time-points. Cytokine expression that occurs earlier (8 h to 48 h pi) compared to later time-points (72 h pi) likely has a greater impact on viral replication (Fig. 5). Reassortant virus replication was attenuated in human dNHBE, a finding consistent with the antiviral IFN profiles. IFN-stimulated genes (ISGs) ISG15, Mx1 and OAS1 were explored (Fig. 8) as these genes also function to regulate virus replication [47]. As predicted from the IFN expression results (Fig. 7), parental and reassortant viruses induced high ISG expression in dNSBE cells at 24 h pi (Fig. 8A) and 72 h pi (Fig. 8B). Interestingly, in dNSBE cells, expression of all three antiviral effectors were substantially higher compared to in dNHBE cells at 24 h pi, and the trend contrasted at 72 hpi indicating that there are kinetic differences in the antiviral response between human and swine respiratory epithelium. By 72 h pi, dNHBE cells exhibited pronounced up-regulation of IFN genes, while IFN expression levels in dNSBEs appeared unaltered between the 24 h and 72 h time-points (Fig. 8A vs. 8B). These anti-viral differences may account for virus replication differences between swine and human cell types.

## Discussion

Identification of reassortment trends between endemic circulating SIVs and seasonal human strains of IAV can provide a framework for identifying potential factors involved in pathogenicity and novel gene constellations that enable zoonosis. The emergence of new influenza A viruses with pandemic potential requires crossing species barriers for transmission to humans. There is evidence that this process is mediated by particular viral genes and their reassortment, in particular, the viral polymerase complex (PA, PB1, PB2). In this study, the ability of polymerase genes to allow reassortant viruses to cross-species barriers was examined in normal fully-differentiated swine and human bronchoepithelial cells because the origin of most pandemic strains appears to originate in swine, which are considered ‘mixing vessels’. Our results show that human-origin PA has a preferential reassortment advantage within a swine influenza virus polymerase complex, but is restricted in human but to a lesser degree in swine respiratory epithelial cells.

To identify features that contribute to viral attenuation, virulence, and novel reassortment of polymerase gene constellations, the reassortment prevalence for six gene segments was analyzed initially in PK-1 cells and subsequently in NSBE cells. The analysis revealed 33% reassortment prevalence in PK-1 cells, and 14% prevalence in NSBE cells. The true number of reassortants is possibly higher as the qPCR assay was limited to six genes, omitting PB1 and NP in the initial multiplex screen. Despite this limitation, the methods used to screen have an advantage over a reverse-genetics system as they allow comparative profiling of the reassortment prevalence after co-infection with parental strains (pdm09 and swH3N2), and is consistent with the natural reassortment of H3N2v in swine. Based on the reassortment profiles, PA, PB1, PB2 and NP from pdm09 contribute to viral fitness, whereas displacement of any of these four genes by a swine H3N2 segment counterpart is not well tolerated. In support are the findings here showing not one reassortant virus being detected having a huH1N1-swPA constellation compared to the common swH3N2-huPA constellation.

The swH3N2-huPA constellation was closely evaluated as PA is essential for polymerase function, and because of the limited knowledge regarding whether the reassortment of PA subunits with different viral origins affects species tropism. Studies examining the PA gene of pdm09 within a swine H3N2 architecture were also pursued based on previous studies reporting that the pdm09 PA within seven genes of highly pathogenic avian influenza (HPAI) H5N1 enabled respiratory droplet transmission in a guinea pig model and enhanced polymerase activity in humans cells [48]. Recent studies have shown that pdm09 PA can also lessen species-specific host restriction within an avian viral polymerase complex in 293T cells to expand tropism [49].

While the PA segment encodes PA-X, an immune modulator, there was no evidence of excess type I or III IFN responses to pdm09 or swH3N2 relative to swH3N2-huPA or swH3N2-huPA-PB1 response in dNHBE cells. These results suggest that pdm09 PA, and/or differences in the NS1 gene, a regulator of the host IFN response, are not exclusively responsible for attenuation of swH3N2-huPA replication and the high levels of IFN gene expression observed. Sequencing of all parent viruses and reassortants did not reveal any genetic mutations to PA and NS segments. It should be noted that the IAV polymerase complex can inhibit activation of the IFNβ promoter independent of NS1 function [50]. It is conceivable that an all-inclusive polymerase cassette of PA, PB1 and PB2 from swH3N2, or pdm09 may be needed to optimally replicate in dNHBE cells without triggering a robust IFN response. pdm09-PA and swH3N2-PB1 and -PB2 polymerase constellations in swH3N2-huPA may conceivably be tolerated to a greater extent in dNSBE cells but not dNHBE cells. Alternatively, acquisition of PB1 in the form of swH3N2-huPA-PB1 may not be tolerated in both swine dNSBE and human dNHBE cells.

In summary, IAV polymerase segments appear to contribute to both reassortment and viral fitness by promoting adaption to cross-species barriers of replication. Changes that affect the host range affect pandemic potential. Based on the findings from this study and others [48], it is clear that pdm09 PA reduces the barrier to species-specific host restriction particularly for the swine pdm09 PA reassortant (swH3N2-huPA). Potential differences in expression of α2–3 or α2–6 sias did not account for the attenuated replication phenotype of swH3N2-huPA or parent virus replication in dNHBE and dNSBE cells. Reassortant or parental virus infection of dNHBE cells was characterized by a pronounced type I and III IFN and ISG antiviral response, while attenuation of virus replication was not evident in the swine-derived dNSBE cells. These findings indicate that differences in the type I and III IFN response dictates in large part cross-species barriers to infection of reassortant viruses. Additionally, the discrepancy between increased lung pathology in ferrets infected with swH3N2-huPA, but apparent attenuation of replication in human cells, is intriguing and suggests additional studies are needed to determine which of these two systems may be translationally more informative. Nonetheless, the studies reported here provide a framework for understanding pandemic potential and host cell features that affect influenza virus reassortment in humans and swine.

## Supporting Information

Figure S1 Primer and probe sequences used in multiplex qPCR and SYBR green qPCR to determine parent viral segments during reassortment studies. Noted in primer sequence is the following: IAbRQsp: Iowa Black Quencher short wavelength emission; IABFQ: Iowa Black far wavelength emission; ZEN: Zen Quencher; Probe: denotes same probe used for both virus gene segments due to high sequence similarity within amplified region, SYBR: SYBR Green qPCR assay used to discern PB1 and NP gene segments of select assortments.
(TIFF)

Figure S2 qPCR multiplex for determining A/Sw/PA/62170-1/2010 [taxid: 938271] and A/California/04/2009 [taxid: 641501] genome segments. A). Plaques were analyzed in one of two 3-plex qPCR assays for each virus to deduce reassortment trends for 6 of the 8 genome segments. qPCR multi-plex dCT values, shown in bold font reveal specificity for 3-plex qPCR assay. See Fig. S1 for specified primer-probe sequences.
(TIFF)

Figure S3 Immunofluorescence analysis of swH3N2-huPA and pdm09 viruses at 48h pi in dNHBE cells. These studies show attenuation of viral spread in dNHBE cells following a starting MOI = 0.01. Blue: DAPI, Green: Influenza NP, Red: α2–6 sialic acid, and merged panels, shown at (A) 20× and (B) 40× magnified view.
(TIFF)
