# Supplementary figures and images for: Polymerase Discordance in Novel Swine Influenza H3N2v Constellations Is Tolerated in Swine but Not Human Respiratory Epithelial Cells
Source: PLoS One. 2014 Oct 16;9(10):e110264. doi: 10.1371/journal.pone.0110264 (PMC4199677; doi:10.1371/journal.pone.0110264)

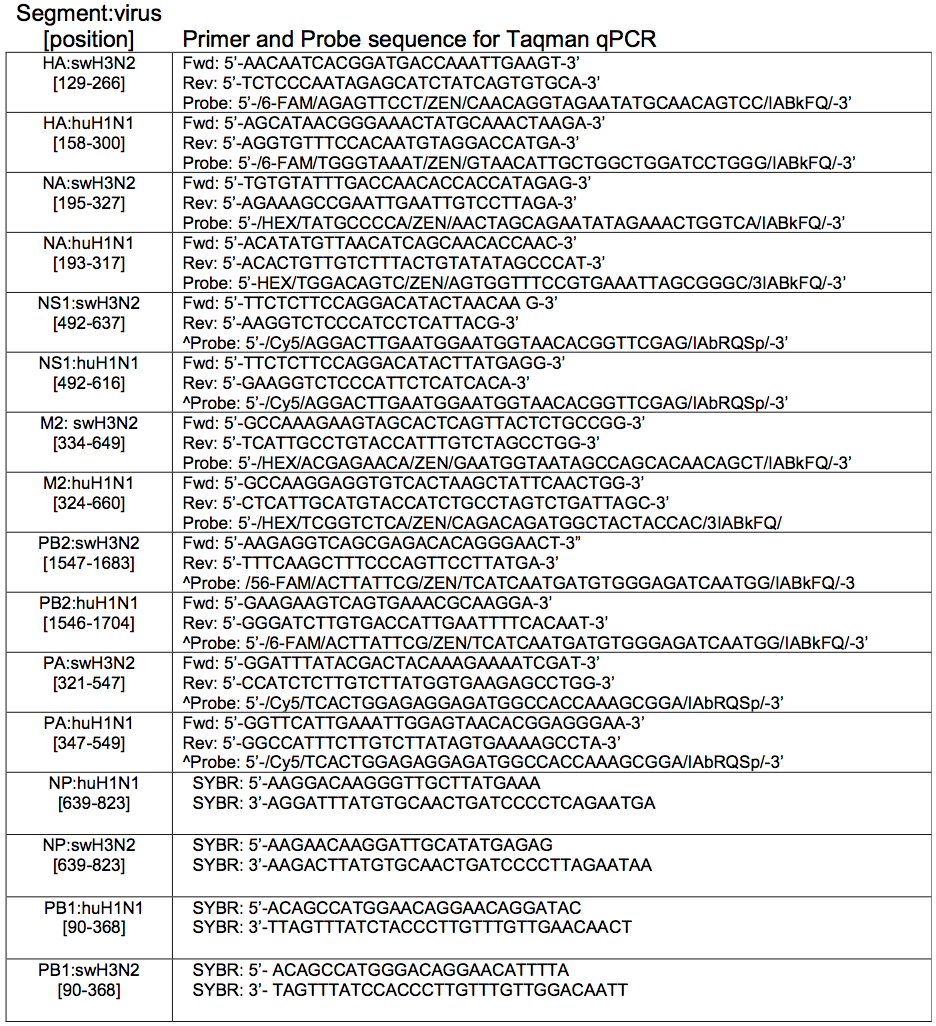

Supplement: Figure S1 — Primer and probe sequences used in multiplex qPCR and SYBR green qPCR to determine parent viral segments during reassortment studies. Noted in primer sequence is the following: IAbRQsp: Iowa Black Quencher short wavelength emission; IABFQ: Iowa Black far wavelength emission; ZEN: Zen Quencher; Probe: denotes same probe used for both virus gene segments due to high sequence similarity within amplified region, SYBR: SYBR Green qPCR assay used to discern PB1 and NP gene segments of select assortments. (TIFF) [file pone.0110264.s001.tiff]

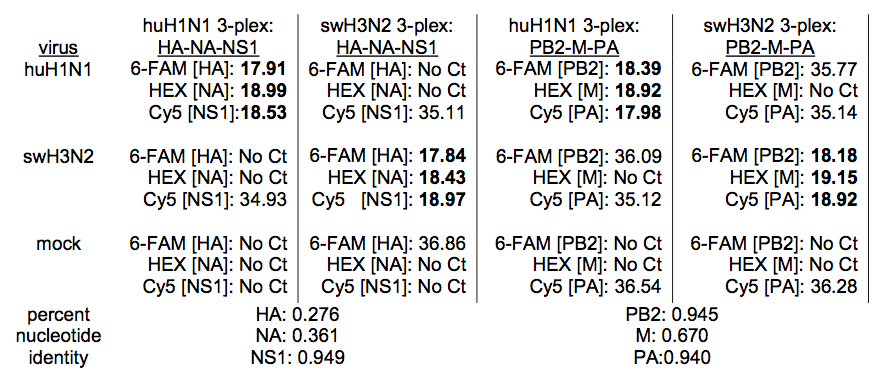

Supplement: Figure S2 — qPCR multiplex for determining A/Sw/PA/62170-1/2010 [taxid: 938271] and A/California/04/2009 [taxid: 641501] genome segments. A). Plaques were analyzed in one of two 3-plex qPCR assays for each virus to deduce reassortment trends for 6 of the 8 genome segments. qPCR multi-plex dCT values, shown in bold font reveal specificity for 3-plex qPCR assay. See Fig. S1 for specified primer-probe sequences. (TIFF) [file pone.0110264.s002.tiff]

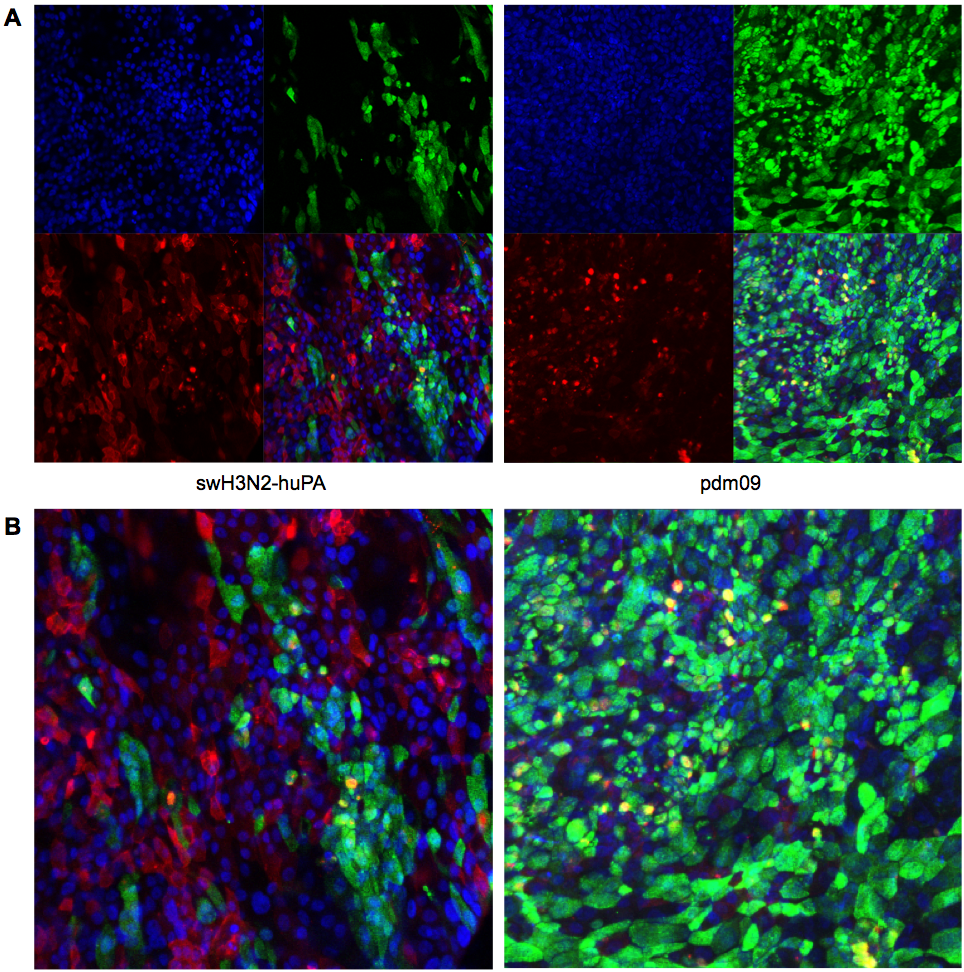

Supplement: Figure S3 — Immunofluorescence analysis of swH3N2-huPA and pdm09 viruses at 48h pi in dNHBE cells. These studies show attenuation of viral spread in dNHBE cells following a starting MOI = 0.01. Blue: DAPI, Green: Influenza NP, Red: α2–6 sialic acid, and merged panels, shown at (A) 20× and (B) 40× magnified view. (TIFF) [file pone.0110264.s003.tiff]
